# Four-state memory based on a giant and non-volatile converse magnetoelectric effect in FeAl/PIN-PMN-PT structure

**DOI:** 10.1038/srep30002

**Published:** 2016-07-15

**Authors:** Yanping Wei, Cunxu Gao, Zhendong Chen, Shibo Xi, Weixia Shao, Peng Zhang, Guilin Chen, Jiangong Li

**Affiliations:** 1 Key Lab for Magnetism and Magnetic Materials of the Ministry of Education, Lanzhou University, Lanzhou, 730000, China; 2 Institute of Chemical & Engineering Sciences, Agency for Science, Technology and Research, Singapore Synchrotron Light Source, National University of Singapore, 5 Research Link, 117603, Singapore

## Abstract

We report a stable, tunable and non-volatile converse magnetoelectric effect (ME) in a new type of FeAl/PIN-PMN-PT heterostructure at room temperature, with a giant electrical modulation of magnetization for which the maximum relative magnetization change (ΔM/M) is up to 66%. The 109° ferroelastic domain switching in the PIN-PMN-PT and coupling with the ferromagnetic (FM) film via uniaxial anisotropy originating from the PIN-PMN-PT (011) surface are the key roles in converse ME effect. We also propose here a new, four-state memory through which it is possible to modify the remanent magnetism state by adjusting the electric field. This work represents a helpful approach to securing electric-writing magnetic-reading with low energy consumption for future high-density information storage applications.

Multiferroic materials are characterized by structures in which their magnetism can be modulated via electric fields in terms of the converse magnetoelectric (ME) effect^1^,^2^. Due to the swiftness, low power consumption, and energy efficiency of said ME effect^3^,^4^, multiferroic material structures are some of the most promising candidates for the next generation of non-volatile, random access memory applications (e.g., sensors and spintronics devices)^5^,^6^. To date, electric field-based magnetism control has been extensively researched experimentally in intrinsic multiferroic materials and artificial ferromagnetic (FM)/ferroelectric (FE) heterostructures^7^,^8^,^9^,^10^, as this allows access to magnetism modulation through electric fields rather than magnetic fields or heavy currents, and thus offers utmost access via electric-writing magnetic-reading for low-energy-consumption memory stores in the post-Moore era^11^,^12^. Single-phase multiferroic materials are rare at room temperature, however, and their intrinsic coupling between magnetization and polarization at the atomic scale is generally weak-characteristics which altogether restrict the realistic utilization of these materials^13^. Recent studies have shown that the exploitation of artificial two-phase systems comprised of FM and FE materials is a helpful technique for achieving electric-field magnetism modulation^14^,^15^,^16^,^17^,^18^,^19^.

As the electric-field magnetism modulator, a giant, stable, tunable, and non-volatile converse ME effect is the key to successful information storage^8^. There exist three main approaches to achieving the desired magnetism modulation:^20^,^21^ intrinsic ME coupling, direct electric field effect, and strain-mediated ME effect. Among these approaches, the strain-mediated converse ME effect is the most efficient on account of its large magnetic variation to electric-filed stimuli^12^. Typically, a reversible, butterfly-like behavior is observed in the strain-mediated FM/FE heterostructures by the bipolar-electric-field modulated magnetization; the change in magnetization is volatile, as the strain disappears when the external electric field is removed^14^,^15^,^16^,^17^,^18^. Therefore, in electric-field magnetism modulation, a non-volatile change in magnetization at room temperature is readily expected. Previous researchers have accordingly attempted to develop non-volatile piezostrain-mediated electric field magnetization control techniques^19^,^22^,^23^,^24^,^25^ and been mildly successful, however, for high-density information storage, a non-volatile, multi-state memory with sufficiently large converse ME by electric impulses in multiferroic material has been elusive. Jiang *et al*. reported a non-volatile, three-state resistance switching technique via electric impulses in converse ME multiferroic heterostructures but reached a maximum relative magnetization change (ΔM/M) of only 4.3%^26^.

FeAl is a magnetostriction material^27^, which shows perfect soft ferromagnetism. Moreover, when considering economic reasons and commercialization, low cost FeAl alloys are the most promising FM candidate. By contrast, yielding ultra-high piezoelectric behaviors, single crystals of lead magnesium niobate-lead titanate (PMN-PT) are generally used for FE^15^,^25^. More recently, due to the higher thermal and electrical stability of the rhombohedral ferroelectric phase compared to binary PMN-PT systems, ternary lead indium niobate (PIN)-PMN-PT has been studied, as it has an approximately 40 °C higher rhombohedral-tetragonal phase transition temperature than that of PMN-PT and maintains the same excellent piezoelectric properties as PMN-PT^28^,^29^,^30^,^31^,^32^. Therefore, to achieve a giant, stable, tunable and non-volatile ME effect, the combination of a FeAl thin film and a PIN-PMN-PT(011) single crystal is a perfect candidate.

In this paper, we report a giant, stable, tunable and non-volatile converse ME effect in a new type of FeAl/PIN-PMN-PT FM/FE heterostructure at room temperature with electrical magnetization modulation; the maximum relative magnetization change (ΔM/M) of the proposed system is up to 66%, i.e., quite high compared to those reported in similar studies^14^,^16^,^26^. We also propose a new four-state memory which can be modified with the remanent magnetism state by adjusting the electric field. We established an illustrative example using the ASCII codes for electric field-based magnetism control in information technology. To our knowledge, this is the first report on controlling multiple states of remanent magnetism in this type of FeAl/PIN-PMN-PT FM/FE heterostructure.

## Results

**Figure 1:**
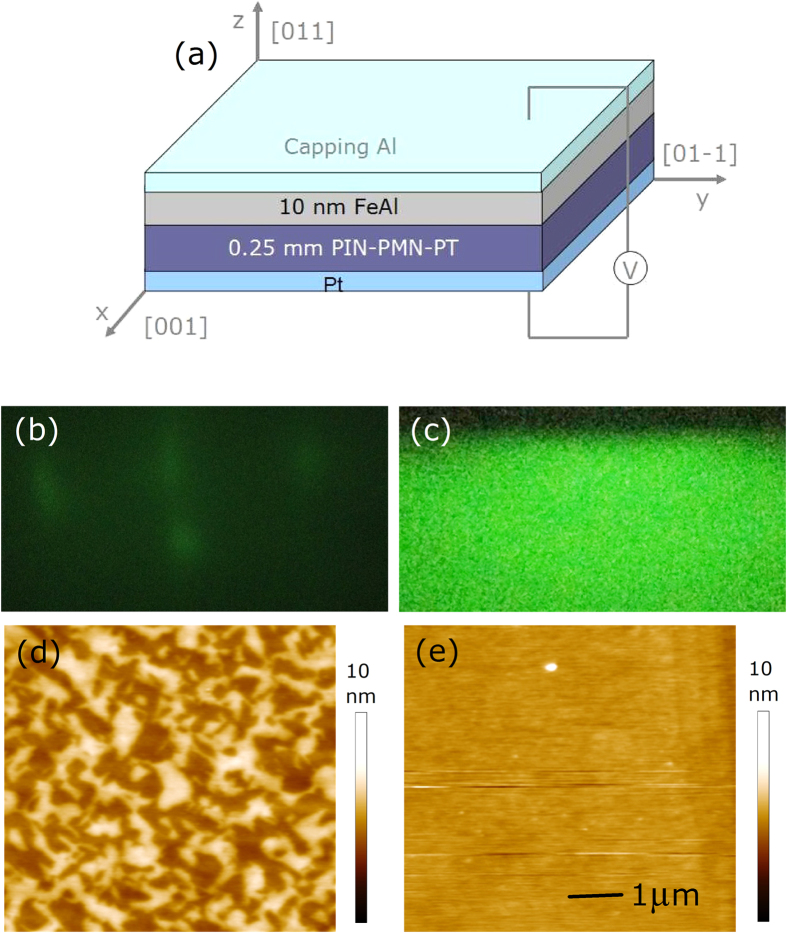
Schematic illustration of the sample and experimental configuration, RHEED patterns and AFM micrographs of the PIN-PMN-PT(011) substrate and Fe_81_Al_19_ film grown on it, respectively. The electron beam is along the in-plane direction of [100].

To construct the FeAl/PIN-PMN-PT heterostructure (Fig. 1a), 10-nm-thick amorphous Fe_81_Al_19_ film were deposited on (011)-oriented PIN-PMN-PT single-crystal substrates via molecular beam epitaxy (MBE). Before observing the converse magnetoelectric effect in the FeAl/PIN-PMN-PT structure, the reflection high-energy electron diffraction (RHEED) patterns and atomic force microscopy (AFM) images of the PIN-PMN-PT substrate and Fe_81_Al_19_ film after deposition was complete were first examined. Figure 1b shows the RHEED pattern of the (011)-oriented PIN-PMN-PT single-crystal substrate. After growing Fe_81_Al_19_, the streak patterns gradually changed to diffusion; finally, no diffraction pattern was observed upon completion of the deposition, as shown in Fig. 1c. This indicates that the amorphous phases of the Fe_81_Al_19_ film are formed on the PIN-PMN-PT substrate. Figure 1d shows an AFM micrograph of the PIN-PMN-PT substrate, with a root-mean-square (rms) roughness of 1.1 nm, which is mostly smooth for deposition. When deposition of the Fe_81_Al_19_ film was complete, the surface exhibited a small rms roughness of 0.5 nm, as shown in Fig. 1e, which is perfect for the next stage of the study.

**Figure 2:**
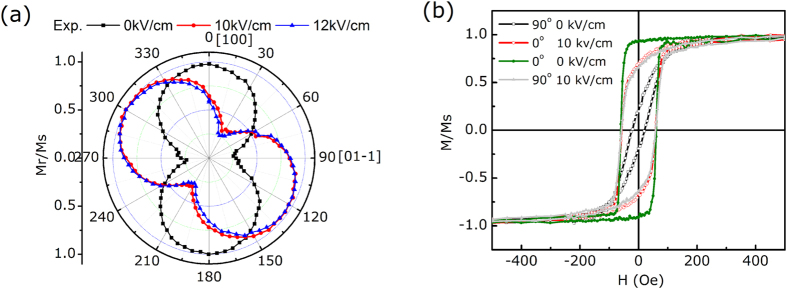
(**a**) The angular dependence of remanent magnetization (*M*_*r*_) under 0 kV/cm, 10 kV/cm and 12 kV/cm. (**b**) In-plane hysteresis loop with different electric fields along 0° and 90°, including magnetization process changes.

To demonstrate the magnetic anisotropy of the FeAl film, angle-remanent curves were measured in different electric fields of 0 kV/cm, 10 kV/cm and 12 kV/cm using a vibrating sample magnetometer (VSM). Before obtaining experimental data, a saturation field of 2000 Oe was applied and then set to zero to achieve a remanence state. Figure 2a shows the angular dependence of remanent magnetization (*M*_*r*_) in different electric fields; here, an angle of 0° is the starting point in our experiment. The 0 kV/cm curve shows a uniaxial anisotropy of ultrathin FeAl film that originates from epitaxial growth on the PIN-PMN-PT(011) surfaces. At 0° and 90°, the *M*_*r*_ reaches its maximum and minimum value, respectively, which indicate the easy magnetizing axis and hard magnetizing axis, respectively, and correspond to the PIN-PMN-PT [100] and [01–1] directions. When applying a dc electric field of 10 kV/cm along the [0–1–1] direction normal to the substrate, an obvious 55° shift in the easy axis direction is observed, which indicates electric field-based control of the magnetism. However, after exceeding 10 kV/cm, almost no changes can be observed, as shown in the 12 kV/cm curve. According to the angle-remanent curves, the magnetic hysteresis loops were measured along the [100] direction at 0° and the [01–1] direction at 90^o^, with electric fields of 0 kV/cm and 10 kV/cm using VSM, respectively. The magnetization process of the sample along the [01–1] direction becomes easy and the *M*_*r*_ increases when the electric field increases, as shown in Fig. 2b. Meanwhile, the magnetization process along the [100] direction is the reverse, with *M*_*r*_ decreasing in the electric field of 10 kV/cm. How these data related to the anisotropic strain of the (011)-oriented PIN-PMN-PT under electric fields will be discussed in the discussion section.

**Figure 3:**
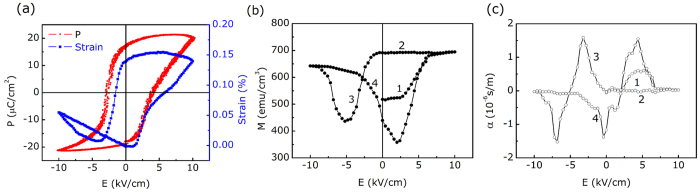
(**a**) Polarization-electric field (P-E) and strain-electric field (S-E) curves of single crystalline PIN-PMN-PT (011). (**b**) Electric field modulation of the in-plane magnetization measured along the [01–1] direction with an external field of 8 Oe. (**c**) The magnetoelectric coefficient α as a function of the electric field obtained from differentiating magnetization-electric field curves.

To study the origin of the variation of magnetization that is due to the electric field, we measured the polarization and piezoelectric strain of (011)-oriented PIN-PMN-PT single crystals as a function of the electric field using a ferroelectric test system and a strain gauge, respectively. As expected, the strain-electric field (S-E) curves in Fig. 3a show a special loop-like shape, which may originate from the 109° ferroelastic domain switching. To clarify the correlation between the variation of magnetization and electric field directly, the variation of magnetization along the [01–1] direction with the electric field was measured at 8 Oe, at which the converse ME effect is more notable. The numbers marked on the curves in Fig. 3b show the variation sequence of magnetization to the electric field, and yield a loop-like M-E curve that matches the S-E curve shape and the intense magnetization process changes that occur near the coercive electric field of PIN-PMN-PT. Obviously, the M-E curve for our FeAl/PIN-PMN-PT FM-FE structure is distinct from previous reports, which exhibited butterfly-like M-E curves and volatile changes of magnetization^14^,^15^,^16^,^17^,^18^. Although in Ga_1−x_Mn_x_As/FE and La_0.8_Sr_0.2_MnO_3_/FE structures, researchers found loop-like M-E curves, the M-E effect in these samples occurred under extreme conditions below −170 °C^33^,^34^. However, in the FeAl/PIN-PMN-PT FM-FE structure, we obtained a giant, stable, tunable and non-volatile converse ME effect at room temperature. Specifically, in the CoFeB/PMN-PT structure reported in ref. 8., the authors attributed the large electric-field-controlled magnetization to the in-plane magnetic isotropy of the FM layer in addition to the strain mediation of the piezoelectric substrates. However, for our FeAl/PIN-PMN-PT structure, we grew the FM layer with an obvious uniaxial anisotropy and even obtained a large electric-field-controlled magnetization ΔM/M of up to 66%. Note that the magnetoelectric coefficient, defined as α = μ_0_dM/dE^2^, can be obtained by differentiating the M-E curve in Fig. 3b. The magnetoelectric coefficient α, as a function of the electric field, is shown in Fig. 3c; the maximum value of α is 1.6 × 10^−6^ s/m, which is 2 orders of magnitude greater than those from the former reports^2^,^14^,^16^. Thus, the tunable converse ME effect reported here is particularly significant in terms of giant and non-volatile magnetization variation.

**Figure 4:**
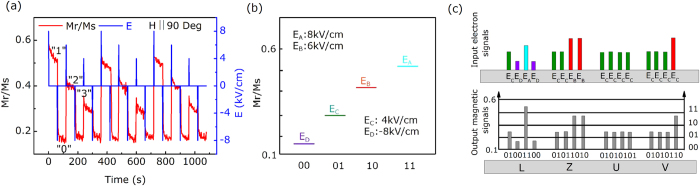
(**a**) The changes of M_r_ along 90° under different pulsed electric fields and the corresponding four-state memory application. (**b**) Demodulation of the two-digit information of “00”, “01”, “10”, and “11” with the corresponding electric fields of −8 kV/cm (E_D_), 4 kV/cm (Ec), 6 kV/cm (E_B_) and 8 kV/cm (E_A_), respectively. (**c**) Coding the word “LZUV” according to the ASCII codes.

Considering the requirements of applications, *M*_*r*_ under different pulsed electric fields was measured. Intermittent positive electric fields of 4 kV/cm, 6 kV/cm and 8 kV/cm and a negative electric field of 8 kV/cm as an “erased electric pulse”, were applied to the FeAl/PIN-PMN-PT FM-FE structure. Here, the electric field of −8 kV/cm also plays the role of the reset voltage. Routinely, a saturation field of 2000 Oe was applied which was then reduced to zero. Figure 4a shows the electric field-induced non-volatile magnetization switching under the various pulsed electric fields along the [01–1] direction, with a giant electrical modulation of magnetization for which the maximum relative magnetization change (ΔM/M) is up to 66%, which is much larger than those found in the previous reports^14^,^16^,^26^. Therefore, four notable and stable magnetization states can be achieved by simply modulating the electric field. The magnetization under the various pulsed electric fields, labeled as “0”, “1”, “2”, and “3”, exhibit obvious multistate properties. In the strategy of utilizing electric-writing magnetic-reading, one of the key components is the converse ME effect device, which is used to convert the electric signals into magnetic signals. For instance, the four items of two-digit information of “00”, “01”, “10”, and “11” can be demodulated and stored from remanent magnetism with the corresponding *M*_*r*_*/M*_*s*_ device as 0.15, 0.3, 0.4, and 0.5 in response to the electric fields of −8 kV/cm (E_D_), 4 kV/cm (Ec), 6 kV/cm (E_B_) and 8 kV/cm (E_A_), respectively, as shown in Fig. 4b. Moreover, according to the ASCII codes and the standard eight-bit codes of “01001100”, the letter “L” can be demodulated and stored in the FeAl/PIN-PMN-PT FM-FE structure. This non-volatile multistate memory through electric-writing magnetic-reading can be written efficiently with lower energy consumption and can store information with higher density compared to the traditional memory methods. The other letters can also be demodulated in the same way. Therefore, the word “LZUV”, which is an abbreviation for Lanzhou University, can be demodulated from the electric field and stored in the FeAl/PIN-PMN-PT FM-FE structure, as shown in Fig. 4c.

## Discussion

**Figure 5:**
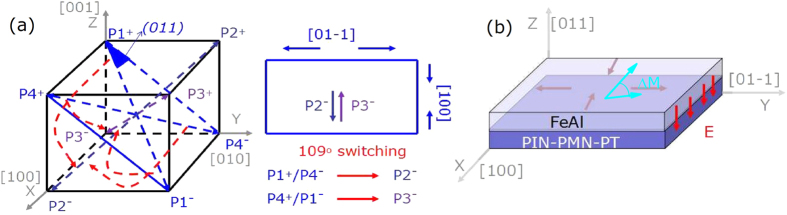
(**a**) Physical model for 109° ferroelastic domain switching under electric fields. (**b**) Configuration for the piezoresponse of the PIN-PMN-PT substrate and a tensile and compressive strain with direction change of easy magnetization.

**Figure 6:**
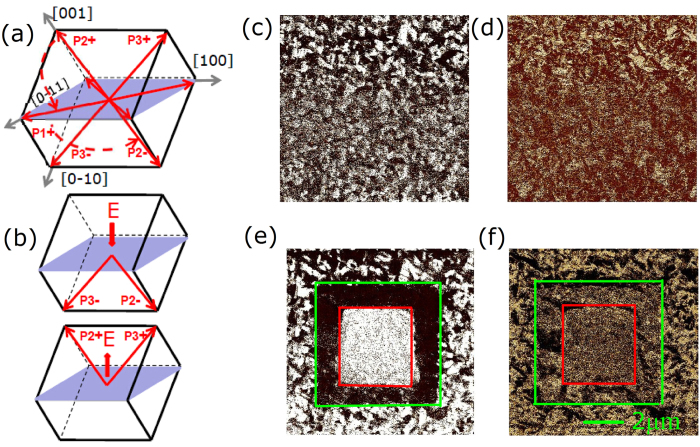
(**a,b**) are the schematics showing the initial, postive poled, and negative poled states of the polar vectors. (**c,e**) are out-of-plane phase PFM image while (**d,f**) are the in-plane PFM phase images.

To illustrate the strain-mediated ME effect, we propose the model shown in Fig. 5. In this work, (011)-oriented PIN-PMN-PT single-crystal substrates are used for FE and the spontaneous polarizations of PIN-PMN-PT with rhombohedral phase are along the <111> directions (Fig. 5a). Upon being exposed to an external positive electric field along the [0–1–1] direction normal to the substrate, all of the eight possible polarization directions are switched downward, except p2^−^ and p3^−^ (Fig. 5a). Larger external electric fields further switch the polarizations p2^−^/p3^−^ toward the [0–1–1] direction, inducing a tensile stress strain along the [01–1] direction and a compressive strain along the [100] direction (Fig. 5b). This causes the magnetization process of the sample along the [01–1] direction to become easy and along the [100] direction to become hard. As shown in Fig. 5a, polarization changes with p1^+^/p4^−^ to p2^−^ or p4^+^/p1^−^ to p3^−^ are related to 109° ferroelastic domain switching, which can remain and result in loop-like behavior for the strain-electric field curve and is the origin of the non-volatile converse ME effect^12^. When an external negative electric field is applied to this heterostructure, once beyond the coercive electric field of PIN-PMN-PT, all of the polarizations return to the initial state and the heterostructure recovers its original condition. This model can explain the loop-like behavior of the S-E curve and the origin of the nonvolatility. We should note that the results cannot be explained by the terms related to the electric-field induced charge accumulation at the interface, because its contribution is too small to account for the achieved large modulation in magnetization which should come from the entire film^33^,^35^. To further discuss the correlations between polarization states and different electric fields in (011)-oriented PIN-PMN-PT, we investigated the substrates with piezoresponse force microscopy (PFM). In non-poled PIN-PMN-PT, the polarization vectors appeared randomly along the eight body diagonals as shown in Fig. 6a. This created the PFM phase images out-of-plane and in-plane shown in Fig. 6c,d, respectively, with the cantilever along the [100] direction. In out-of-plane measurement, the tip was sensitive to the vertical piezo response and the PFM phase images had two kinds of color contrast. while in-plane measurements, the tip was sensitive to component polarization perpendicular to the cantilever besides out-of-plane polarization and, so there were three kinds of color contrast in the PFM phase images. After applying a voltage of +10 V on the tip along the [0–1–1] direction normal to the substrate (green box) with a 5 μm by 5 μm square (Fig. 6e), polarization switching with a two-step switching sequence composed of a 71° switch (e.g., P2^+^ changes to P1^+^) followed by a 109° (e.g., P1^+^ changes to P2^−^) switch took place and finally changed to P2^−^ and P3^−^, thus the color of the out-of-plane image became black and the color of the in-plane image bacame brown. Accordingly, the in-plane polarization had no component along the [0–11] direction and thus there was no contrast within the green box in Fig. 6f. When a voltage of −10 V was applied along the [0–1–1] direction normal to the substrate (red box) within a 3 μm by 3 μm square, the polarization directions were switched conversely upward apart from p2^+^ and p3^+^, thus the out-of-plane image became white (Fig. 6e). The in-plane polarization again had no component along the [0–11] directions, so there is no contrast changes within the red box as shown in Fig. 6f. The stable and reversible in-plane and out-of-plane polarization states, including 109° ferroelastic domain switching, enable non-volatile converse ME effect.

**Figure 7:**
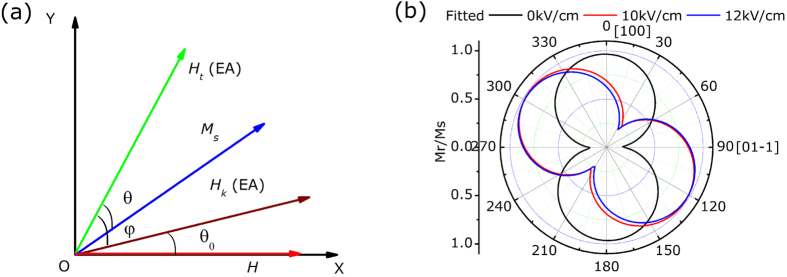
(**a**) Schematic of the coordinate system. (**b**) Calculated angular dependence of *M*_*r*_.

Finally, we calculated the angular dependence of *M*_*r*_ to understand the experimental shift of anisotropy. The total energy *E*_*t*_ is written according to the Stoner-Wohlfarth model^36^


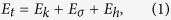


where *E*_*k*_ is the magnetic anisotropy energy, *E*_*σ*_ is the stress energy and *E*_*h*_ is the Zeeman energy. Here, we define *E*_*u*_ as the total anisotropy energy, which consists of *E*_*k*_ and *E*_*σ*_. Figure 7a shows the coordinate system for the analysis; *θ*_*0*_ is the angle between the directions of the applied magnetic field *H* and the as-deposited anisotropy field *H*_*k*_; *φ* is the angle between the directions of the total effective field *H*_*t*_ after applying a dc electric field and *H*_*k*_; *θ* is the angle between the directions of *H*_*t*_ and *M*_*s*_. In the actual measurement of VSM, *H*_*k*_ is not always along the x-axis and *θ* is always 0. In this case, the total energy can be expressed as


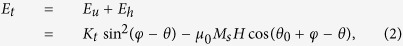


where *K*_*t*_ is the in-plane total effective uniaxial anisotropy constant. *M*_*r*_, achieved in the angle-remanent curves, satisfies


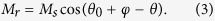


The equilibrium conditions can be written as


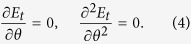


Because *M*_*r*_ is always positive and a differential constant C, we can obtain


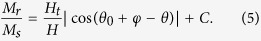


Based on formula (5), fitted curves of *M*_*r*_ vs *θ*_*0*_are shown in Fig. 7b, which agree well with the experimental data. When applying an electric field, the easy axis is driven to a new stable direction due to the total effective field *H*_*t*_. Namely, the angle *φ*, fitted from Fig. 2a, in formula (5) determines the easy magnetizing direction under various electric fields. This indicates that the total effective anisotropy field *H*_*t*_ has switched by nearly 55^o^ from the as-deposited *H*_*k*_ after applying the 10 kV/cm electric field; the calculation result agrees well with the experimental shift in anisotropy.

In summary, a stable, tunable and non-volatile converse ME effect is obtained in a new type of FeAl/PIN-PMN-PT FM/FE heterostructure at room temperature with a giant electrical modulation of magnetization. Test results show that the 109^o^ ferroelastic domain switching in PIN-PMN-PT and coupling with FM film with uniaxial anisotropy originated from the surface of PIN-PMN-PT (011) were the key factors in the converse ME effect observed. Owing to the giant electrical modulation of magnetization, four-state memory through electric-writing magnetic-reading can meet the application requirements of high-density information storage, which allows demodulation from the electric field and can be stored in the FeAl/PIN-PMN-PT FM-FE structure. This work is helpful for exploring the basic principles of electric-field modulation of magnetism and applications, especially electric-writing magnetic-reading, to achieve low energy consumption for future high-density information storage.

## Methods

10-nm-thick amorphous Fe_81_Al_19_ film were first grown at RT on PIN-PMN-PT(011) substrates using MBE equipped with solid-source effusion cells for Fe and Al, at a rate of 0.13 nm/min and 0.33 nm/min for Fe and Al respectively. Then, a capping layer of 3 nm of Al was grown on the top of the sample to prevent the FeAl film from oxidizing and also it could be used as a top electrode. Prior to growth, the substrate was chemically cleaned using trichloromethane, acetone, and methanol. After the substrate was loaded into the growth chamber, the substrate was further thermally cleansed at an annealing temperature of 100 °C. Nucleation and growth were monitored *in situ* using RHEED. Pt layers were finally sputtered as the bottom electrode via magnetron sputtering at room temperature. The surface morphologies of substrates and samples were examined using AFM. In the magnetoelectric coupling test, Cu varnished wires were connected to the electrode by adhesive tape and the electric field applied between the top and bottom electrode was controlled by the dc power supply Keithley 6517B. The polarization and piezoelectric strain of (011)-oriented PIN-PMN-PT single crystals, as a function of the electric field, were measured using a ferroelectric test system (Radiant, Precision Premier II, USA) and a strain gauge (KFG-1–120-D17, Kyowa), respectively. The ferroelastic domain switching in the PIN-PMN-PT under electric field was observed using PFM.

## Additional Information

**How to cite this article**: Wei, Y. *et al*. Four-state memory based on a giant and non-volatile converse magnetoelectric effect in FeAl/PIN-PMN-PT structure. *Sci. Rep.* **6**, 30002; doi: 10.1038/srep30002 (2016).
